# Focused maternity care in Ghana: results of a cluster analysis

**DOI:** 10.1186/s12913-016-1654-5

**Published:** 2016-08-17

**Authors:** Martin Amogre Ayanore, Milena Pavlova, Wim Groot

**Affiliations:** 1 Department of Health Services Research, CAPHRI, Maastricht University Medical Centre, Faculty of Health, Medicine and Life Sciences, Maastricht University, Maastricht, The Netherlands; 2 Top Institute Evidence-Based Education Research (TIER), Maastricht University, Maastricht, The Netherlands; 3 Centre for Health Policy, Advocacy, Innovation & Research in Africa (CHPAIR-Africa), Accra, Ghana

**Keywords:** Delivery care, Facility utilisation, Medication care, Prenatal care, Postnatal care, Skilled delivery, WHO delivery care components, Ghana

## Abstract

**Background:**

Ghana missed out in attaining Millennium Development Goal 5 in 2015. The provision of adequate prenatal and postnatal care remains problematic, with poor evidence on women’s views on met and unmet maternity care needs across all regions in Ghana. This paper examines maternal care utilization in Ghana by applying WHO indicators for focused maternal care utilization.

**Methods:**

Two-step cluster analysis segregated women into groups based on the components of the maternity care used. Using cluster membership variables as dependent variables, we applied multinomial and binary regression to examine associations of care use with individual, household and regional characteristics.

**Results:**

We identified three patterns of care use: adequate, less and least adquate care. The presence of a female and skilled provider is an indicator of adequate care. Women in Volta, Upper West, Northern and Western regions received less adequate care compared with other regions. Supply-related factors (drugs availability, distance/transport, health insurance ownership, rural residence) were associated with adequacy of care. The lack of female autonomy, widowed/divorced women, age and parity were associated with less adequate care. Care patterns were distinctively associated with the quality of health care support (skilled and female attendant) instead of with the number of visits made to the facility. Across regions and within rural settings, disparities exist, often compounded by supply-related factors.

**Conclusions:**

Efforts to address skilled workforce shortages, greater accountability for quality and equity, improving women motivation for care seeking and active participation are important for maternity care in Ghana.

**Electronic Supplementary Material:**

The online version of this article (doi:10.1186/s12913-016-1654-5) contains supplementary material, which is available to authorized users.

## Introduction

Ghana introduced a fee-exemption policy for maternity care in 2007 to accelerate the progress towards the achievement of Millennium Development Goals (MDGs) 4 and 5. This was also aimed at improving the maternity care utilization. This and other health policies are closely tied to the first population policy of 1969 (revised in 1994) and rooted in the conviction of providing conditions for safe motherhood as a pre-requisite for ensuring prosperity and economic growth. The country launched a new health policy 2007 dubbed “*creating wealth through health”* with a vision to attain a middle-income status by 2015 [1]. With a renewed focus on improving human capital and achieving the MDGs, targeted interventions to scale up high impact and rapid delivery health interventions were stepped up. Efforts at improving health surveillance and accountability to ensure improved health access and quality, received multi-sectorial recognition [1]. These interventions combined with earlier efforts culminated in lowering maternal mortality from 410 per 100,000 live births in 2010 to 380 per 100,000 live births in 2013 [2]. Overall life expectancy improved slightly from 58 years in 2004 to 61 years in 2012 while the country attained a middle-income status in the recent years [3].

Despite these gains, the level of childbirths assisted by a skilled attendant (physician, midwife/nurse or community health officer) in 2008 was low (59 % of all deliveries) compared with the level of antenatal care utilization (95 % of pregnant women) [4]. Recent evidence indicates that an estimated 74 % of national deliveries are attended by a skilled provider [5]. Additionally, maternal and infant mortality rates (380 per 100,000 live births and 52 per 1,000 live births respectively) remain at unacceptable levels. Despite maternal and child friendly center campaigns, and the expansion of Community-based Health Planning and Services (CHPS), many births remain unattended by skilled professional care. Many broader social determinants of health care are found to have a great impact on women maternity care utilization in poor settings [6, 7].

Previous studies in Ghana on maternity care investigated the low care quality and travel distance, skilled provider distributions, out-of-pocket payments for maternity care when insurance is absent, facility type (public/private) and delivery status [8–12]. Empirical evidence also enumerates patient inequalities, provider type relations, socio-economic determinants, visits and utilization effects on neonatal deaths [13–16]. These studies however, analyze users of maternal care as a single group, and provide trends over a specific period. None of these studies provides a segmentation of women’s met and unmet care needs based on care components received according to international standards, e.g. WHO guidelines for focused maternity care [17, 18]. Such segmentation is important for understanding the standards of care met and unmet. This is critical for examining the maternity care delivery packages for improved utilization in settings where skilled professional care is low.

This study examines the components of antenatal and postnatal care utilization in Ghana at three levels; pre and postnatal care, facility utilization and medication care at last birth. By using data from the 2008 Ghana Demographic Health Survey (GDHS) dataset, and employing a cluster and regression analysis, the study offers evidence on the adequacy of care received (adequate, less adequate or least adequate) by different groups, and associated reasons for these variations. The study provides a “*window of opportunity*” to examine the critical salient factors affecting women needs when seeking maternity care. This will inform policy decisions at all levels; district, regional and national for improved maternity care at the primary level for women in Ghana.

## Background

Current interventions in Ghana for safe motherhood aim to improve emergency obstetric care, family planning, and surveillance systems for maternal mortality monitoring and reporting. Other interventions involve the use of novel techniques (High impact Rapid Delivery) to scale up the most targeted interventions [19]. Notwithstanding, within urban and rural areas, maternity care cannot be dissociated from the broader challenges of the general health care system. In a baseline study in 2005, it was shown that access and utilization were inadequate for both basic and emergency obstetric care [19]. Inequality in access to skilled delivery exists. An estimated 36 % of women have access to public facilities while 9 % has access to private facilities. Rural, educational and geographical variations account for this. Earlier national policy directions recognized the role of Traditional Birth Attendants (TBAs), but the recent policy focus has centered on three types of professionals: physicians, midwives/nurse and community health staff. Current data (GDHS, 2008) suggest that only 10 % of women are attended by a physician and 41 % are attended by midwife/nurse during delivery. A significant policy progress was the introduction of a fee-exemption policy and its subsequent take over under the national health insurance scheme to remove financial barriers for care. The assurance of all essential components of maternity care is vital to guarantee a continuum of care for women during and beyond pregnancy (see Table 1).

**Table 1:** Components of WHO “focused care” and Ghana success maternity interventions

*Components of Maternity Care*
In Ghana, maternity care developments are grounded in the WHO guidelines for a “focused antenatal care” for all expectant mothers, including: identifying and managing all pregnancy complications, identifying and treating all concurrent illness whiles taking steps to avoid the complication exacerbation through prevention and case management of anemia and intermittent treatment for malaria (IPTp). Others such as micronutrient supplementation, two doses of tetanus immunizations, education and sensitization on HIV/AIDS, healthy lifestyle behaviors and the monitoring of very vital signs during every stage of pregnancy, are provided. Assisted delivery by a professional at a facility that has the capacity to provide the needed care for a good pregnancy outcome is also essential. Adequate postnatal care is characterized by the presence of skilled care during and for the first 42 days after delivery. Appropriate postnatal check up on the mother and newborn critically depends on receipt of post check after delivery, timing of care received (at least within 1 week for hard to reach areas) and the provider type (skilled or unskilled) providing care. Although antenatal care alone cannot significantly impact maternity outcomes, its adequate provision provides an entry point for integrated care, contributes to skilled utilization at birth and links community care structures with women. Maternity care is delivered in Ghana in both private and public facilities, encompassing all categories of maternity homes, clinics, CHPS compounds, health centers and hospitals. Most maternity homes, clinics (all levels), health centers and regional hospitals exist in urban areas while CHPS centers, health centers or district hospitals if they do exist, provide care for those living in rural areas. Evidencebased approaches to improve delivery outcomes exist in Ghana, such as the community participatory approach (Zorko initiative), Amansie west, Koforidua, and Tamale teaching hospitals experiences. Understanding continuously women in changing demands and needs influencing critical care components for maternity is important to make current strategies and interventions relevant.

Source: 1, 4–6

## Methods

The 2008 GDHS dataset was used in this study. The GDHS is a nationally representative survey that applies standardized data collection instruments to collect information to inform health policy planning and implementation across all 10 regions in Ghana. The 2008 GDHS was implemented by the Ghana Statistical Service and Ghana Health Service with technical support from ICF Macro and MEASURE DHS program [4]. Specifically, a two stage-point cluster and systematic sampling was applied. The first stage sampling was made up of 412 clusters selected from an updated master sampling frame constructed from the 2000 Ghana population and housing census. A listing operation was conducted between June and July 2008 in all selected clusters to provide the sampling frame for the selection of households in the second stage of clustering. Table 2 provides an overview of the household’s distribution and enumeration areas per region. The second stage sampling applied a stratified, two-stage cluster design. A systematic sampling approach selected the households listed in each cluster. The initial objective was to have 30 households per cluster. However, a weighting adjustment procedure was applied according to the population distribution in the 2000 census. Also, one cluster was excluded at the time of data collection due to security reasons. This provided a final sample of 12,323 households nationwide [4]. A total of 11,778 occupied households were interviewed during the 2008 GDHS with a response rate of 96.5 %. The sample selection procedure and the response rate enable separate estimates for respondents in all regions, as well as for rural and urban areas.

**Table 2:** Households distribution and enumeration areas in the 2008 GDHS

Region	Percent distribution households 2000 census	Percent distribution households 2008 GDHS	Total number of Enumeration areas (EAs)
Upper East	3.9	6.8	28
Upper West	2.2	7.3	30
Northern Region	6.6	9.2	38
Brong Ahafo Region	9.3	9.2	38
Ashanti Region	18.4	16.3	67
Eastern Region	12.3	10.4	43
Volta Region	9.3	8.5	35
Greater Accra Region	16.9	14.6	60
Central Region	9.9	8.2	34
Western Region	11.1	9.5	39
Total	100	100	412

Source: Data reported in the 2008 GDHS report

Women aged 15+ years were eligible to be interviewed if they were usual residents or visitors present in the household the night before the interview. In total, 4,916 eligible women were interviewed in the survey. This was made up of 2,162 urban and 2,754 rural eligible women. From them, 2,147 women (763 urban and 1,384 rural women) within reproductive age (aged 15–49 years) were included in our analysis because they reported childbirths within the last 5 years.

### Ethical considerations

All eligible women participants provided informed consent for the study. There was no deception of respondents and no attempts were made to cajole respondents into agreeing to something to which they may not have otherwise agreed. All information that may compromise anonymity of respondents was not retained on record [20]. To use the dataset, we obtained a written approval by ICF Macro and MEASURE DHS, who conducted the initial data collection.

### Dataset variables assessed

The content of the questionnaire in the 2008 GDHS study was based on model questionnaires developed by MEASURE DHS and the 2003 GDHS questionnaire. Field pre-testing was carried out between June and July 2008. The questionnaire was translated from English into three main working languages: Akan, Ewi and Ga. The main data collection was carried out between September and November 2008 involving face-to-face interviews. From the detailed individual women questionnaire, three groups of relevant response variables related to pregnancy and postnatal care based on the WHO indicators as exemplified in Table 1, were assessed;

1. Group 1 containing questions on prenatal care during pregnancy, including 15 questions on prenatal care use, attendant availability (skilled/unskilled) during antenatal visits, pregnancy complication awareness, knowledge and awareness of HIV/AIDS during pregnancy.
2. Group 2 containing questions on facility utilization during pregnancy and after delivery, including 6 questions to assess facility utilization during pregnancy and delivery regarding frequency and place of utilization, as well as health checks before and after discharge.
3. Group 3 containing questions on prenatal and postnatal pregnancy medications received, including 10 questions centered on prenatal and postnatal pregnancy medications received as part of prenatal and postnatal care.

The women in our study sample were asked to provide the above information for their last childbirth during the last 5 years. The English wording of the questions is given in Additional file 1: Appendix B.

### Data analysis

Cluster analysis, a multivariate statistical technique for grouping cases of data, was applied in our analysis. The purpose was to categorize women’s responses into groups or clusters based on the patterns of similarities and dissimilarities of material care received during the last birth. The large sample size coupled with the mixture of categorical and continuous data required the application of a two-step cluster approach instead of other clustering approaches, such as hierarchical and k-means cluster analysis [21]. The two-step clustering procedure allows for an exploratory identification of natural cases/objects within the large dataset. Differences among variables are determined by the log-likelihood distance measure.

The cluster analysis was carried out per group of questions (see data collection above) and for all questions together, resulting in 4 separate clustering procedures (software package SPSS 22). To avoid the exclusion of respondents due to a high rate of missing values (>10 %) in few variables, a category “no response” was created for these variables. Related to reliability and validity, the cluster analysis was repeated several times checking whether the cluster range remained fair to good. The clustering stage was ended when stable cluster membership groups were obtained. All clusters were generated automatically (no manual pre-selection of the number of the clusters) and produced fair to good cluster range (see Additional file 1: appendix C for cluster rangers). The 4 clustering procedures mentioned above, produced 4 cluster membership variables (see Figs. 2, 3, 4 and 5). These membership variables were used as dependent variables in a subsequent binary/multinomial logistic regression analysis depending on the number of the clusters. For all regression models, the explanatory variables included the same set of individual and household socio-demographic characteristics, as well as regional characteristics. The significance threshold was *p <* .05.

## Results

In total, 2147 eligible women respondents who reported childbirths 5 years prior to the study were included in our final analysis. Descriptive statistics of the response and socio-demographic variables of respondents are presented in Additional file 1: Appendix A and B. The results of the cluster analyses (based on the response variables) are first presented in this section, followed by the results of the regression analyses using the cluster membership variables as dependent variables and the socio-demographic characteristics as explanatory variables (absolute value of correlation between explanatory variable < 0.5).

### Cluster findings

**Fig. 1:**
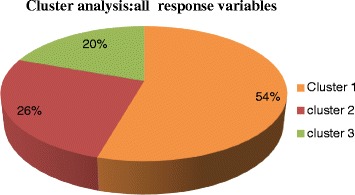
Overall distribution of respondents in the cluster analysis. Note: Cluster 1 (adequate care), Cluster 2 (less adequate care) and Cluster 3 (least adequate worse care)

**Fig. 2:**
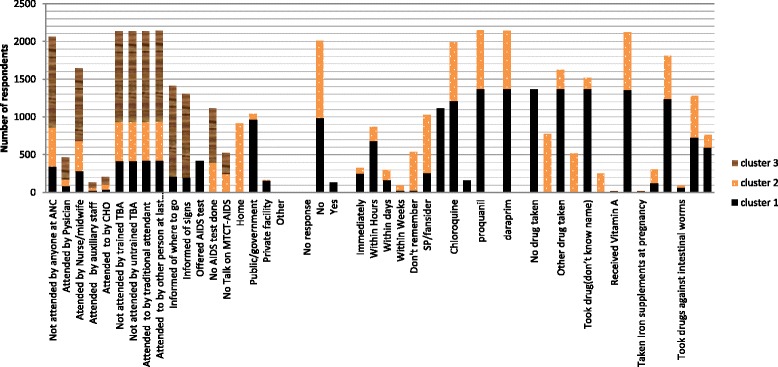
Overall levels of care components received by women (15–49 years). Note: Cluster 1 (adequate care), Cluster 2 (less adequate care) and Cluster 3 (least adequate care)

We first present the results of the two-step cluster procedure based on all response variables (see Additional file 1: Appendix B). Three distinct clusters with varied care components were generated. The cluster group distribution among the three clusters is presented in Fig. 1. Overall, cluster group 1 can be categorized as a group that received adequate care (more skilled attendants, more information about signs of pregnancy complications, and more medication) compared with cluster groups 2 and 3 (see Fig. 2).

From Figs. 1 and 2, we see that cluster group 2 received less adequate care compared to cluster group 1 but overall better care compared to cluster group 3. Cluster group 3 received the least adequate care compared to the other two cluster groups. Details about the cluster patterns and quality are found in the supplementary file labeled Additional file 1: Appendix C. Specifically, compared to cluster groups 2 and 3, women in cluster group 1 were more likely to be attended by a physician, nurse/midwife and auxiliary staff, to have received more information about pregnancy signs and complications, received mother-to-child transmission (MTCT) of AIDS talks, offered AIDS test at ANC, tested for AIDS, delivered at public/government facility, delivered by caesarean, received quick health check-ups after delivery, and received delivery checks before discharge. Other distinctive features of cluster 1 included: more drugs against malaria and intestinal parasites, and postnatal vitamin A and iron supplements during prenatal and postnatal periods. Drugs against malaria for group 1 respondents were more likely to be SP/malafan/fansider, chloroquine and proquanil. Absence of any attendance was less likely in cluster group 1 and early visits for their first antenatal check-up was more likely compared to cluster group 2. However, antenatal visits for cluster group 1 were less likely than for cluster groups 2 and 3.

Women in cluster group 2 were most likely to be attended by a TBA. Compared to cluster groups 3, this group was more likely to have been attended by someone at ANC and was more informed about where to go with pregnancy complications, although these rates were lower than in cluster group 1. Women in cluster group 2 were also more likely to be informed on signs of pregnancy complications, offered an AIDS test and tested for AIDS, and provided talks on MTCT of AIDS compared with group 3, but less likely compared with group 1. Difficulties to recall the timing of the first postnatal check and delivery in other place (than those specifically listed) were more common among cluster group 2 respondents compared to cluster group 1 and 3. Women who took no drugs for malaria or could not recall what drug were taken, were also more likely to belong to cluster group 2 compared to cluster group 3, but less compared to cluster group 1. The majority of women in cluster group 2 (70.0 %) did not take drugs against any intestinal parasites, which was a higher non-use than in group 1 and 3.

Postnatal vitamin A and iron supplement intake was more likely for cluster group 2 respondents compared to cluster group 3. Both cluster 2 and 3 reported less vitamin A intake compared to cluster group 1. Antenatal visits for cluster group 2 was however more likely than for cluster group 1 and less compared to cluster group 3. The early timing of women first antenatal visit was most likely in cluster group 1, less in cluster group 2 and least in cluster group 3. Cluster group 3 women were most likely to be attended to by a TBA (trained/untrained). A high share of this cluster group also reported not being attended by someone/anyone, not being offered AIDS test nor tested for AIDS, and being less likely to have talks on MTCT of AIDS compared with groups 1 and 2. Home delivery was most likely in cluster group 3 compared with cluster groups 1 and 2. Post-delivery check-ups in cluster group 3 were reported to take place within one or more weeks on average. Health checks before discharge after delivery for cluster group 3, were comparable with cluster group 2 but less likely compared with cluster group 1. The use of SP/malafan/fansider and chloroquine as a malaria prevention was reported by cluster group 3, but less often compared to cluster group 1. Women in cluster group 3 were more likely not to take any drug against malaria or recall the name of malaria drugs taken during pregnancy. This was less reported among cluster group 2 and least among cluster group 1. Vitamin A intake and iron recipients for cluster group 3 were comparable with cluster group 2, but less compared with cluster group 1. Cluster group 3 recorded most visits for antenatal check-ups compared with cluster groups 1 and 2, but this group was less likely to have attended earlier first antennal care compared with cluster groups 1 and 2.

**Fig. 3:**
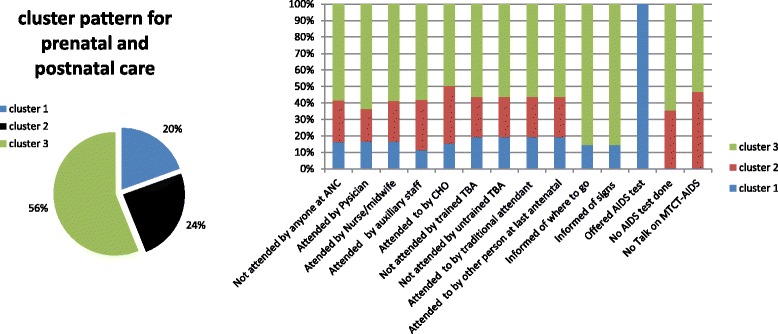
Prenatal and postnatal care cluster characteristics for women (15–49 years). Note: Cluster 1 (adequate care), Cluster 2 (less adequate care) and Cluster 3 (least adequate care)

**Fig. 4:**
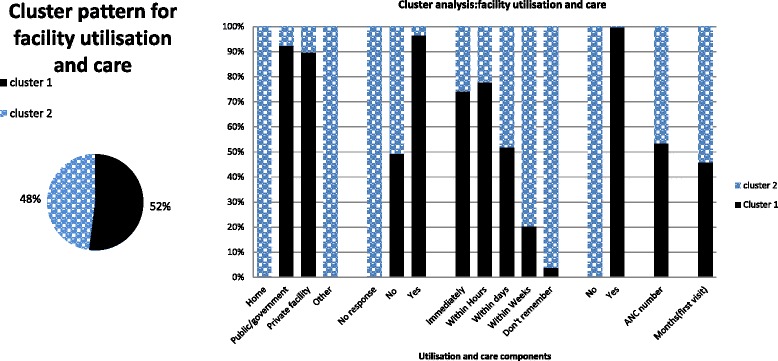
Facility utilisation and other care components received by women (15–49 years). Note: Cluster 1 (adequate utilization care), Cluster 2 (less adequate utilization care)

**Fig. 5:**
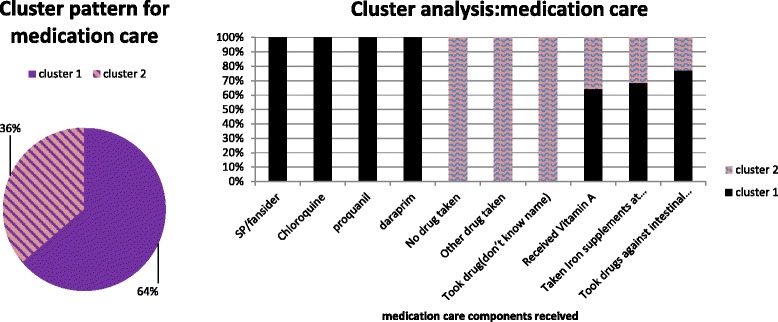
Medication care components received by women respondents (15–49 years). Note: Cluster 1 (adequate medication care), Cluster 2 (less adequate medication care)

To confirm the results of the cluster analysis that included all response variables, we also present the results of three separate cluster analysis performed per group of questions using the two-step cluster approach (see Figs. 3, 4 and 5). The results are comparable to those presented in the overall cluster analysis. Specifically, Figs. 3, 4 and 5 show the proportion of women respondents clustered under each cluster category in the subsequent cluster analyses.

### Results of the multinomial and regression analysis

Regression results are presented here to ascertain the associations between the adequacy of care received by women during pregnancy and childbirth and socio-demographic characteristics. We present the results of four regression analyses (multinomial and binary) where the cluster membership variables generated by the four cluster analyses are used as dependent variables respectively. An identical set of explanatory variables is used as independent variables in all four regressions (see Table 3). In the first regression model, where the cluster membership is based on all response variables, two geographical regions (Volta and Upper West) appeared to have received significantly less adequate care. Also, the likelihood of receiving less adequate care was more for women within the poorest wealth quartile (OR = 1.754, C.I 0.999–3.078). The likelihood of receiving least adequate care was significantly associated with women without health insurance, and those who never slept under insecticide treated nets (ITNs). Additionally, women’s age was also significantly associated with receiving the least adequate care (with older women more frequently receiving least care). The odds of receiving least care was 2.3 times higher in the absence of a female provider at the point of care provision (*p <* 0.003, 95 % C.I 1.319–3.955) and 2.0 times higher for widowed/divorced women (*p <* 0.005, 95 % C.I 1.244–3.371). However, concerns of no drugs available for treatment, difficulty with access (distance/transport), residence place (rural) were also significantly associated with least adequate care received.

**Table 3:** Regression results of cluster membership groups with individual, household and national explanatory variables

Covariates/Socio-demographic	All response variables Adequate care group (ref)		Prenatal and postnatal care Adequate care (ref)		Facility utilization Adequate utilization group (ref)	Medication received Adequate medication care group (ref)
	Multinomial logistic regression				Binary logistic regression	Binary logistic regression
	less adequate care group	least adequate care group	less adequate care group	least adequate care group	less adequate utilization care group	less medication care group
	Exp B (95 % CI)	Exp B (95 % CI)	Exp B (95 % CI)	Exp B (95 % CI)	Exp B (95 % CI)	Exp B (95 % CI)
Age (years)	0.973 (0.951–0.997)*	1.078 (1.052–1.105)***	1.089 (1.063–1.116)***	1.003 (0.980–1.028)	0.971 (0.950–0.993)*	0.973 (0.953–0.992)*
Parity (# of living children)	1.079 (0.987–1.179)	0.919 (0.838–1.008)	0.899 (0.820–0.986)*	1.014 (0.927–1.110)	1.148 (1.055–1.248)**	1.085 (1.005–1.171)*
Rural residence type	1.082 (0.789–1.484)	1.596 (1.124–2.267)*	1.744 (1.229–2.473)*	1.482 (1.075–2.044)*	1.981 (1.490–2.633)***	1.050 (0.795–1.387)
Currently employed	0.855 (0.621–1.178)	0.939 (0.647–1.362)	0.944 (0.651–1.370)	0.847 (0.614–1.167)	0.943 (0.691–1.285)	0.918 (0.691–1.220)
Marital status						
Never married	0.670 (0.389–1.154)	0.998 (0.533–1.868)	1.240 (0.657–2.340)	1.264 (0.756–2.112)	0.859 (0.518–1.425)	0.899 (0.560–1.443)
Widowed/Divorced	1.160 (0.699–1.927)	2.048 (1.244–3.371)*	1.807 (1.107–2.951)*	0.818 (0.483–1.385)	1.193 (0.753–1.891)	1.382 (0.911–2.097)
Married (ref)						
Wealth status						
Poorest	1.754 (0.999–3.078)*	1.042 (0.559–1.946)	0.959 (0.515–1.789)	1.403 (0.793–2.481)	6.300 (3.593–11.046)***	1.601 (0.981–2.614)
Poorer	1.362 (0.813–2.281)	1.042 (0.590–1.842)	1.080 (0.611–1.907)	1.491 (0.888–2.504)	3.175 (1.894–5.324)***	1.205 (0.769–1.888)
Middle	1.313 (0.810–2.130)	1.076 (0.633–1.828)	1.086 (0.640–1.845)	1.391 (0.853–2.268)	2.359 (1.440–3.866)**	1.082 (0.711–1.646)
Richer	1.314 (0.843–2.046)	1.192 (0.737–1.929)	1.065 (0.660–1.720)	0.838 (0.524–1.339)	1.634 (1.015–2.632)*	1.013 (0.689–1.490)
Richest (ref)						
Respondent education						
No education	1.832 (0.738–4.548)	1.468 (0.598–3.604)	1.392 (0.570–3.400)	2.029 (0.720–5.717)	2.336 (0.840–6.496)	1.803 (0.846–3.844)
Primary	1.373 (0.556–3.394)	1.241 (0.510–3.020)	1.374 (0.567–3.329)	2.357 (0.841–6.603)	1.918 (0.692–5.315)	1.349 (0.636–2.862)
Secondary	1.194 (0.495–2.877)	1.068 (0.453–2.518)	1.136 (0.484–2.665)	1.856 (0.678–5.083)	1.373 (0.503–3.751)	1.091 (0.526–2.263)
Higher (ref)						
Region						
Upper East	1.359 (0.735–2.513)	1.260 (0.619–2.564)	1.078 (0.535–2.171)	0.977 (0.514–1.856)	0.919 (0.493–1.711)	1.133 (0.659–1.947)
Upper West	0.364 (0.200–0.663)**	0.793 (0.418–1.502)	0.841 (0.445–1.589)	0.413 (0.224–0.761)*	0.899 (0.509–1.586)	0.387 (0.231–0.649)***
Northern	1.315 (0.772–2.240)	1.418 (0.779–2.580)	1.658 (0.913–3.011)	2.022 (1.166–3.506)*	2.266 (1.312–3.914)*	1.230 (0.773–1.958)
Brong Ahafo	0.854 (0.498–1.465)	0.915 (0.488–1.717)	0.947 (0.506–1.773)	0.903 (0.519–1.573)	0.846 (0.486–1.472)	0.793 (0.491–1.279)
Ashanti	0.839 (0.515–1.366)	0.881 (0.511–1.519)	0.936 (0.544–1.612)	0.992 (0.600–1.641)	1.103 (0.669–1.817)	0.796 (0.519–1.222)
Eastern	0.753 (0.428–1.325)	1.021 (0.553–1.887)	0.999 (0.542–1.841)	0.668 (0.371–1.203)	1.102 (0.633–1.919)	0.730 (0.446–1.195)
Volta	0.347 (0.190–0.635)**	0.745 (0.401–1.384)	0.754 (0.407–1.396)	0.309 (0.162–0.590)***	0.879 (0.510–1.517)	0.457 (0.275–0.760)*
Central	0.674 (0.379–1.197)	0.637 (0.337–1.204)	0.633 (0.336–1.191)	0.617 (0.337–1.127)	1.678 (0.968–2.908)	0.701 (0.426–1.156)
Western	1.226 (0.707–2.123)	1.098 (0.589–2.048)	0.951 (0.513–1.764)	0.784 (0.440–1.398)	2.136 (1.227–3.718)*	1.276 (0.789–2.062)
Accra (ref)						
Partner education						
No education	0.982 (0.755–1.277)	1.100 (0.823–1.470)	1.124 (0.842–1.500)	1.073 (0.817–1.409)	1.121 (0.876–1.434)	1.041 (0.827–1.312)
Primary	1.104 (0.696–1.751)	0.979 (0.556–1.722)	1.034 (0.589–1.815)	1.343 (0.842–2.142)	0.822 (0.529–1.277)	1.104 (0.729–1.671)
Secondary	0.678 (0.318–1.445)	0.665 (0.258–1.716)	0.833 (0.320–2.166)	1.323 (0.648–2.702)	0.724 (0.368–1.422)	0.795 (0.406–1.558)
Higher (ref)						
Covered by NHIS	0.851 (0.669–1.083)	0.592 (0.450–0.780)***	0.592 (0.450–0.780)	0.851 (0.669–1.083)***	0.508 (0.406–0.637)***	0.848 (0.686–1.049)
Concerned that no drugs available	0.657 (0.423–1.021)	0.591 (0.364–0.962)*	0.591 (0.364–0.962)	0.657 (0.423–1.021)	1.048 (0.694–1.583)	0.796 (0.544–1.164)
Slept under ITNs	0.872 (0.697–1.090)	0.503 (0.389–0.650)***	0.503 (0.389–0.650)	0.872 (0.697–1.090)***	0.962 (0.776–1.193)	0.861 (0.707–1.049)
Final say on own health						
Respondents	1.065 (0.791–1.434)	1.310 (0.948–1.811)	1.310 (0.948–1.811)*	1.065 (0.791–1.434)*	0.929 (0.700–1.234)	1.133 (0.874–1.468)
Husband/partner	1.197 (0.919–1.558)	1.168 (0.862–1.583)	1.168 (0.862–1.583)***	1.197 (0.919–1.558)	1.024 (0.793–1.323)	1.124 (0.889–1.422)
Both spouses (ref)						
Difficulty with night/day vision at pregnancy						
Big problem	0.958 (0.681–1.349)	0.734 (0.477–1.130)	0.734 (0.477–1.130)	0.958 (0.681–1.349)	1.208 (0.865–1.687)	0.909 (0.666–1.242)
Not a big problem	1.315 (0.960–1.801)	1.369 (0.971–1.930)	1.369 (0.971–1.930)	1.315 (0.960–1.801)	1.131 (0.836–1.530)	1.262 (0.959–1.660)
No problem (ref)						
Difficulty with distance/transport on health needs						
Big problem	0.941 (0.632–1.401)	1.250 (0.819–1.907)	1.250 (0.819–1.907)	0.941 (0.632–1.401)	1.027 (0.706–1.494)	0.986 (0.702–1.386)
Not a big problem	0.792 (0.596–1.053)	0.671 (0.486–0.928)*	0.671 (0.486–0.928)	0.792 (0.596–1.053)	0.712 (0.543–0.934)*	0.766 (0.596–0.983)*
No problem (ref)						
Difficulty with money/permission on getting health						
Big problem	0.890 (0.593–1.333)	1.207 (0.727–2.003)	1.207 (0.727–2.003)	0.890 (0.593–1.333)	1.036 (0.683–1.570)	0.874 (0.607–1.259)
Not a big problem	0.723 (0.473–1.105)	1.290 (0.764–2.179)	1.290 (0.764–2.179)	0.723 (0.473–1.105)	0.835 (0.543–1.284)	0.784 (0.536–1.147)
No problem (ref)						
No provider/female provider available						
Big problem	0.953 (0.689–1.317)	1.378 (0.944–2.012)	1.378 (0.944–2.012)	0.953 (0.689–1.317)	1.086 (0.798–1.479)	1.037 (0.778–1.383)
Not a big problem	1.543 (0.949–2.507)	2.284 (1.319–3.955)*	2.284 (1.319–3.955)	1.543 (0.949–2.507)*	0.869 (0.550–1.375)	1.348 (0.883–2.057)
No problem (ref)						
Regression statistics						
*observations*	2147		2147		2147	2147
*–2LL Ratio*	3939.823		3879.066		2270.445	2604.792
*–Pseudo R square*	0.176		0.179		0.372	0.127
*–P values*	0.000		0.000		0.217	0.407

(ref)-all reference categories included in analysis *** *p* = 0.000, ** *p* ≤ 0.001, * *p* ≤ 0.0

The regression model based on membership variables generated by the three cluster analyses groups showed similar results. Close associations related to regional variations and wealth status were observed in these three regressions as well. Similar close associations were also found to relate to respondents’ parity and autonomy (final say on health). Regarding prenatal and postnatal care and information received, parity and respondents’ own say on their health needs had a positive association with receiving least adequate care. All other factors associated with the least adequate care (women without health insurance, and those who never slept under ITNs, no female provider, widowed/divorced and residence type) were similar to those reported above. The probability of receiving least adequate care was 1.7 times higher for rural compared to urban residents (*p =* 0.002 95 % C.I 1.229–2.473). However, Volta, Upper West and Northern regions also showed close associations with less adequate care. The odds ratio for women living in the Northern region to receive less adequate care was twice higher compared with the other nine regions. Also, the influence of male partner on woman’s health status was positively associated with women who reported less adequate care. The odds of less adequate care was greater for women who expressed the absence of a female provider at the point of service use (OR = 2.284 95 % C.I 1.319–3.955). Female autonomy (final say on women health) was positively related with less adequate care. A woman’s place of residence had a positive association with less adequate care.

Wealth status (decreasing wealth) had a strong positive association with less facility utilization. Women within the poorest wealth quartile were 6 times more likely to utilize less care (*p =* 0.000, 95 % C.I 3.593–11.046). Women in the Northern and Western regions expressed low facility utilization levels. Rural women were most likely to report less utilization of facilities compared with urban residents. High parity levels and women without health insurance were associated with less utilization. Higher parity was positively associated with less facility utilization, less medication care as well as worse care observed for all individual cluster membership groups. Poor access factors (distance/transport) and two regions (Northern and Western) showed strong associations with lower utilization levels. Overall, age, parity, regional variations, residence type (rural/urban) and not covered by health insurance were observed as the most significant factors with a greater propensity to influence care in all membership groups. Respondents and their partner educational status had no significant associations with less adequate or least adequate care received. Other factors, such as employment status and women reported difficulties in getting money for care, showed no significance for the least care observations recounted among women.

## Discussion

In this study, we examined maternal care utilization components in Ghana based on the 2008 GDHS dataset. Individual, household and regional factors were used as explanatory variables to ascertain possible differences between those who experienced adequate care and those who received less or least adequate care. More than half of women in our sample received adequate prenatal and postnatal care. Worrying is the equally approximate share of women who received less and least adequate care. Nevertheless, even among those who used adequate care, there were some drawbacks in the utilization of the recommended focus care components as overall antenatal utilization was low compared with less and least adequate care groups. This study specifically establishes that the frequency of antenatal visit is not associated with the adequacy of care received. Indirect cost and poor access, mediated through health insurance and travel cost, impact greatly on focused maternity care in Ghana. The results also show inequities across the regions with regard to the adequacy of care received, emphasizing that optimal maternal care at a national level was not attained in Ghana.

### Overall prenatal and postnatal care experiences

Our cluster analysis findings reinforced that women who were attended by skilled personnel were more likely to report adequate care irrespective of the frequency of antenatal visits. Adequate care was observed in cluster patterns related to skilled-provider presence and type (male/female), instead of the number of visits to the facility, reaffirming that the frequency of visits did not guarantee good care. Overall less adequate care was attributable to wealth and regional variations. Volta, Upper West and Northern regions had more women reporting less adequate care. The evidence from these regions for less adequate care shows regional variations in service utilization in Ghana. The Northern region was observed to have the least adequate care in Ghana. The Northern and Western regions showed lower levels of facility use and medication care. According to the 2008 GDHS report, the Northern region had the highest fertility rate of 6.8 in Ghana. Poor access and increasing parity as well as service quality and variations between rural and urban areas could have accounted for differences in these four regions (Volta, Upper West, Northern and Western). Although wealth status did not impact directly on the least adequate care received, its indirect impact elicited by the lack of public health insurance, was evident as a strong positive associate for least adequate care. The lack of health insurance contributing to least adequate care could have resulted in a decline in facility visits since direct out-of-pockets payments would create financial obstacles to seek care. This association is supported by evidence that women with public health insurance are likely to improve utilization of health care services [22–26]. The concern of no drugs and women’s inability to use ITNs contributed to the least adequate care, providing corroborations with earlier findings in Ghana that health interventions such as sleeping under ITNs (child and pregnant woman) are not wealth manifested, although they remain significant to bridge inequities for maternal and child health outcomes [26]. Women’s recount of drugs unavailability resulting in least adequate care is supported by previous maternity care findings that supply driven factors regulated by weak health delivery systems reduces the quality of maternity care for most women [27–29].

We found that geographic factors arbitrated by rural residence and access (transport/distance) impact prenatal and postnatal care. This corroborates the existing evidence across Ghana that a woman’s rural context impacts on maternity and reproductive care utilisation [30–32]. There is also overwhelming evidence to support these factors as mediators for safe maternity care in Ghana and across developing countries [7, 11, 29, 33–35]. Although regional variations were not observed for rural residence and access factors (transport/distance), older women and widowed/divorced women experienced less or least adequate care. The absence of a female provider was not a problem in seeking maternity care, but it was reported as a significant consideration, with no observed regional variation. However, these women were more likely to be living in rural areas.

Our finding that increasing age and parity are associated with less or least adequate care is well established in several studies [22, 28, 36–38] as these women are likely to assume confidence in themselves for their own maternity needs. This could be evident in environments where cost and access factors (distance/transport) may create a condition of “despair” and lower seeking behavior for them. Women own say on health was positively associated with least adequate care in this study. This is surprising in the light of health equity campaigns to improve self-autonomy for women health needs. Evidence of poor health care seeking behavior of women attributed to their perceived low self-worth and lack of independence on health-related decisions is reported in most developing countries [33, 39–41]. Additionally, Eliason et al. (2013) affirms that aside autonomy, women personal conviction is important in the utilization of maternal post-partum health services [27]. It may suggest that, female autonomy alone may be illusive for improving maternity care if “enabling factors” such as those that provide an effective feedback mechanism for poor access and utilization, and those that seek to economically liberate women are not in congruence with equity and autonomy campaigns. Efforts at addressing the domineering ideological perception of the male spouse as the determinant of womanhood across many socio-cultural, political and social structures are also important. Educational status of women and male partners had no positive association with less and least adequate care in this study.

An evidenced limitation to this study is the recall bias as a result of women recount of events during their last birth. The dataset does not provide the possibility to corroborate the women’s responses by using data provided by other family relatives or local health professionals. However, our method of cluster analysis provided a good agglomeration of responses which yielded a fair cluster quality on which this study preceded. Since the data were cross-sectional accounts from the 2008 GDHS dataset, causality of prenatal and postnatal care with these associations must be interpreted with care. Due to limitations related to the content of the dataset, we also acknowledge that we might have missed other possible factors that have affected women final responses for maternity care use. Also, we might have missed the experience of some women who only had stillbirths or miscarriages in the preceding 5 years because this is a culturally-sensitive topic and such events are usually underreported in surveys. Regional and place of residence variations and trends for maternity care were not explored in this study since we were only interested in segregating results for possible variables providing less and least adequate care for women. Also, the data that we analyzed come from a survey conducted in 2008 which was the only available dataset at the time of analysis. A similar analysis based on new or earlier GDHS waves can provide a base for a comparison across the years.

### Policy implications for maternity care in Ghana

Health insurance provides potential benefits for prenatal and postnatal facility utilization especially among rural residents. Improving structural deficits under the current national health insurance scheme will inject more confidence for users, and non-active members who have lost out due to issues of inefficiency and poor quality. We suggest the revision of the insurance type and benefits packages, with target premiums that enable pro-poor households to access not only maternity care, but a continuum of care that indirectly impacts women’s health during birth. Current capitation reforms under the health insurance must integrate value-based health care that seeks to provide focused maternity and respond to the general health service need among vulnerable population groups. Additionally, efforts at improving service delivery modes concurrently at the individual, community and facility levels through skilled attendants (physician, midwives, CHO) availability, service provision through increased trainings, motivation and strict deployment particularly for rural areas are necessary. There is a need to develop and enforce strict policy guidelines and monitoring to ensure skilled personnel equity between rural and urban areas. A direct policy option to attract and retain skilled personnel to rural areas is needed. Efforts at rolling out the CHPS concept nationwide are long overdue and must be expedited quickly.

Government’s policies such as fee exemption for maternity care suffered financial deficits and challenges after initial donor funding from the Dutch government elapsed. Evidence suggests that these policies are often poorly implemented with structural deficits and much political influence that does not create accountability for the beneficiaries [42–44]. We suggest long term financial planning with a coherent maternity care policy plan that creates greater accountability for quality, equity and geography (region and rural residence) where the CHPS system may not have reached.

Lastly, broadening and expanding the opportunities for women beyond having a say on their health needs can help alienate socio-cultural values that devalue women. Incorporating sustained behavior communication strategies into training curricula for health staff will enable community health workers target and design effective maternity adoptive health behaviors at the facility and community. Older and multiparous women need targeting at the community level to improve their urgency for service utilization.

## Conclusions

Our study re-emphasizes that close to half of women in Ghana experienced less than adequate maternal care considering three aspects of care: prenatal and postnatal care, facility utilization and medication care. Skilled female provider presence enhances adequate care for women at maternity. Across regions and within rural settings, disparities exist, often compounded by supply-related factors. Efforts at addressing skilled workforce shortages, greater accountability for quality and equity, and those that improve women urgency, choices and active participation are important to improve maternity care in Ghana. The cluster methodology in this study has enabled us to segment components of care received. It remains important to design mechanisms to identify those with less and least adequate care use for effective and targeted interventions that improve safe motherhood initiatives in Ghana. It is also important through these mechanisms to identify geographical (regional and place of residence) differences that exist in care levels and groups of women needing safe, effective and respective care. The study advocates long term planning and systemic reforms on health insurance and maternity care policies. Improving health systems, skilled personnel allocation for care, health staff curricula trainings that recognizes behavior change communication strategies elements for health trainees and broadening women opportunities are vital to the total process of womanhood care beyond 2016.

## Additional file

Additional file 1: Single and group cluster model summaries and cluster quality of membership variables. (DOCX 152 kb)

## Abbreviations

ANC: Antenatal care
CHO: Community health officer
CHPS: Community-based health planning and services
DHS: Demographic health survey
GDHS: Ghana demographic health survey
GHS: Ghana health service
IPT: Intermittent preventive treatment
ITNs: Insecticide treated nets
MDG: Millennium development goals
MTCT: Mother-to-child transmission
TBA: Traditional birth attendant
