# Supplementary material for: Focused maternity care in Ghana: results of a cluster analysis
Source: BMC Health Serv Res. 2016 Aug 17;16:395. doi: 10.1186/s12913-016-1654-5 (PMC4989378; doi:10.1186/s12913-016-1654-5)

**APPENDIX A**

**Table 1: Individual household and health status socio-demographics of respondents**

|  | **Responses**  **Valid(N)**  **Missing(0)** | **Category N (%)** | **Mean** | **S.D**  **(S.E of mean)** | **Variance** |
| --- | --- | --- | --- | --- | --- |
| Age | Valid(2147)  missing(0) | Below 19 105(4.9%)  20-39 1770(82.4%)  40-49 272(12.7%) | 30.20 | 7.277  ( 0.157) | 52.949 |
| Parity | Valid (2147)  missing(0) | 0 16(0.7%)  2-3 983(45.8%)  3-4 659(30.7%)  5+ 489 (22.8%) | 3.12 | 1.939  (0.042) | 3.762 |
| Current employed | Valid (2147)  missing(0) | No 280(13.0%)  Yes 1867(87.0%) | 1.8696 | 0.33684  (0.00727) | 0.113 |
| Marital status | Valid (2147)  missing(0) | Never married 117(5.4%)  Married 1991(92.7%)  Widowed 39(1.8%) | 1.96 | 0.267  (0.006) | 0.071 |
| Partner  education | Valid (2147)  missing(0) | No education 1219(56.8%)  Primary level 127(5.9%)  Secondary level 44(2.0%)  Higher 757 (35.3%) | 2.16 | 1.405  (0.030) | 1.975 |
| Wealth index | Valid (2147)  missing(0) | Poorest 635(29.6%)  Poorer 472(22.0%)  Middle 375(17.5%)  Richer 390(18.2%)  Richest 275(12.8%) | 2.6265 | 1.39939  (0.03020) | 1.958 |
| Region | Valid (2147)  missing(0) | Western 189(8.8%)  Eastern 158(7.4%)  Central 210(9.8%)  Volta 181(8.4%)  Upper west 187(8.7%)  Ashanti 318(14.8%)  Brong Ahafo 207(9.6%)  Northern R. 306(14.3%)  Upper East 181(8.4%)  Accra 210(9.8%) | 5.7420 | 2.75862  (0.05954) | 7.610 |
| Residence  type | Valid (2147)  missing(0) | Urban 763(35.5%)  Rural 1384(64.5%) | 1.6446 | 0.47874  (0.01033) | 0.229 |
| Educational status- | Valid (2147)  missing(0) | No education 774(36.1%)  Primary level 508(23.7%)  Secondary level 817(38.1%)  Higher 48(2.2%) | 2.0647 | 0.90921  (0.01962) | 0.827 |

**Table 1: Individual household and health status socio-demographics of respondents (*continued*)**

|  | Responses  **Valid(N)**  **Missing(0)** | Category N (%) | Mean | S.D  (S.E of mean) | Variance |
| --- | --- | --- | --- | --- | --- |
| Difficulty_  Night/day  vision | Valid (2147)  missing(0) | No problem 1627(75.8%)  Big problem 230(10.7%)  Not big problem 290(13.5%) | 0.3773 | 0.71086  (0.01534) | 0.505 |
| Getting healthcare-  Distance/  transport | Valid (2147)  missing(0) | No problem 1627(75.8%)  Big problem 230(10.7%)  Not big problem 290(13.5%) | 1.3465 | 0.88061  (0.01901) | 0.775 |
| Getting healthcare  money_  permission | Valid (2147)  missing(0) | No problem 1627(75.8%)  Big problem 230(10.7%)  Not big problem 290(13.5%) | 1.4061 | 0.64393  (0.01390) | 0.415 |
| Getting healthcare  No/  female provider | Valid (2147)  missing(0) | No problem 1627(75.8%)  Big problem 230(10.7%)  Not big problem 290(13.5%) | 1.3340 | 0.79477  (0.01715) | 0.632 |
| Covered  NHIS | Valid (2147)  missing(0) | No 1249(58.2%)  Yes 898(41.8%) | 0.4183 | 0.49339  (0.01065) | 0.243 |
| Concern-  no drugs  available | Valid (2147)  missing(0) | Big problem 230(10.7%)  Not big problem 290(13.5%) | 1.5370 | 0.49874  (0.01076) | 0.249 |
| slept under  net | Valid (2147)  missing(0) | No 1168(54.4%)  Yes 979(45.6%) | 0.4560 | 0.49817  (0.01075) | 0.248 |
| Final Say on own  health | Valid (2147)  missing(0) | Respondent 439(20.4%)  Husband/partner 888(41.4%)  Both spouses 820(38.2%) | 2.1775 | 0.74510  (0.01608) | 0.555 |

***NHIS-refers to the National social health insurance scheme in Ghana***

**APPENDIX B**

**Table 2: Results of cluster analysis: prenatal care during pregnancy**

|  | Cluster 1 | Cluster 2 | Cluster 3 | Total |
| --- | --- | --- | --- | --- |
|  | N=419 | N=524 | N=1204 | N=2147 |
|  | n (%) | n (%) | n (%) | n (%) |
| Attended to by no one during the last antenatal period   - No response - No - Yes | 2(0.48%)  335(79.95%)  82(19.57%) | 0(0.0%)  524(100%)  0(0.0%) | 0(0.0%)  1204(100%)  0(0.0%) | 2(0.09%)  2063(96.09%)  82(3.82%) |
| Attended to by a physician   - No response - No - Yes | 2(0.48%)  340(81.15%)  77(18.38%) | 0(0.0%)  431(82.25%)  93(17.75%) | 0(0%)  908(75.42%)  296(24.58%) | 2(0.09%)  1679(78.20%)  466(21.70%) |
| Attended to by nurse/midwife   - No response - No - Yes | 2(0.48%)  143(34.13%)  274(65.39%) | 0(0.0%)  118(22.51%)  406(77.48%) | 0(0.0%)  236(19.60%)  968(80.39%) | 2(0.09%)  497(23.15%)  1648(76.76%) |
| Attended to by auxiliary staff   - No response - No - Yes | 2(0.48%)  401(95.70%)  16(3.82%) | 0(0.0%)  482(91.98%)  42(8.02%) | 0(0.0%)  1123(93.27%)  81(6.73%) | 2(0.09%)  2006(93.43%)  139(6.47%) |
| Attended to by CHO   - No response - No - Yes | 2(0.48%)  385(91.89%)  32(7.64%) | 0(0.0%)  451(86.07%)  73(13.93%) | 0(0.0%)  1101(91.45%)  103(8.55%) | 2(0.09%)  1937(90.22%)  208(9.69%) |
| Attended to by trained TBA   - No response - No - Yes | 2(0.48%)  413(98.57%)  4(0.95%) | 0(0.0%)  521(99.43%)  3(0.57%) | 0(0.0%)  1197(99.42%)  7(0.58%) | 2(0.09%)  2131(99.25%)  14(0.65) |
| Attended to by untrained TBA   - No response - No - Yes | 2(0.48%)  412(98.33%)  5(1.19%) | 0(0.0%)  518(98.85%)  6(1.15%) | 0(0.0%)  1200(99.67%)  4(0.33%) | 2(0.09%)  2130(99.21%)  15(0.69%) |
| Attended to by village volunteer   - No response - No - Yes | 2(0.48%)  417(99.52%)  0(0.0%) | 0(0.0%)  524(100%)  0(0.0%) | 0(0.0%)  1197(99.42%)  7(0.58%) | 2(0.09%)  2138(99.58%)  7(0.33%) |
| Attended to by traditional attendant   - No response - No - Yes | 2(0.48%)  415(99.01%)  2(0.48%) | 0(0.0%)  520(99.24%)  4(0.76%) | 0(0.0%)  1200(99.67%)  4(0.33%) | 2(0.09%)  2135(99.44%)  10(0.47%) |
| Attended to by other person at last antenatal   - No response - No - Yes | 2(0.48%)  416(99.28%)  1(0.24%) | 0(0.0%)  521(99.43%)  3(0.57%) | 0(0.0%)  1203(99.92%)  1(0.08%) | 2(0.09%)  2140(99.67%)  5(0.23%) |
| Informed where to go with any complications   - Don’t know - No - Yes | 85(20.29%)  127(30.31%)  207(49.40%) | 1(0.19%)  523(99.81%)  0(0.0%) | 0(0.0%)  0 (0.0%)  1204 (100%) | 86(4.01%)  650(30.27%)  1411(65.72%) |
| Informed about signs of pregnancy complications   - Don’t know - No - Yes | 212 (50.59%)  17 (4.06%)  190(45.35%) | 524(100%)  0 (0.0%)  0(0.0%) | 1 (0.08%)  86(7.14%)  1117(92.77%) | 737(34.32%)  103(4.79%)  1307(60.88%) |
| Offered AIDS test as part of ANC visits   - Don’t know - No - Yes | 0(0.0%)  0 (0.0%)  419 (100%) | 367(70.04%)  157 (29.96%)  0(0.0%) | 639(53.07%)  565 (46.93%)  0(0.0%) | 1006(46.86%)  722(33.63%)  419(19.52%) |
| AIDS test done during ANC visits   - Don’t know - No - Yes | 415(99.05%)  4 (0.95%)  0 (0.0%) | 0(0.0%)  392 (74.81%)  132 (25.19%) | 0(0.0%)  721(59.88%)  483(40.11%) | 415(19.33%)  111752.03%)  615(28.65%) |
| Talk on MTCT – AIDS received   - Don’t know - No - Yes | 417 (99.52%)  0 (0.0%)  2(0.48%) | 0(0.0%)  248(47.33%)  276(52.67%) | 0(0.0%)  281(23.34%)  923(76.66%) | 417(19.42%)  529(24.64%)  1201(55.94%) |

(Appendix B *continued*)

**Table 3: Results of cluster analysis: facility utilization**

| Variables | Cluster 1 | Cluster 2 | Total |
| --- | --- | --- | --- |
|  | N=1116 | N=1031 | N=2147 |
|  | n (%) | n (%) | n (%) |
| Place of delivery   - Home - Public/government facility - Private facility - Other | 0(0.0%)  962(86.20%)  154(13.79%)  0(0.0%) | 919(89.14%)  83(8.05%)  18(1.75%)  11(1.07%) | 919(42.80%)  1045(48.67%)  172(8.01%)  11(0.51%) |
| Was delivery by caesarean section   - No response - No - Yes | 0(0.0%)  983(88.08%)  133(11.92%) | 2(0.19%)  1024(99.32%)  5(0.48%) | 2(0.09%)  2007(93.48%)  138(6.43%) |
| Timing of first check after delivery   - Immediately - Within hours - Within days - Within weeks - Don’t remember | 244(21.86%)  676(60.57%)  157(14.07%)  20(1.79%)  19(1.70%) | 86(8.34%)  196(19.01%)  147(14.26%)  79(7.66%)  523(50.73%) | 330(15.37%)  872(40.61%)  304(14.16%)  99(4.61%)  542(25.24%) |
| Health check before discharge   - No - Yes | 0(0.0%)  1116(100%) | 1026(99.52%)  5(0.48%) | 1026(47.79%)  1121(52.21%) |
|  | Mean (SD) | Mean (SD) | Mean (SD) |
| Number of ANC visits at pregnancy | 3.8369(0.54sd) | 3.3763(0.98sd) | 3.6157(0.82) |
| Months before first ANC visit | 1.3961(0.54sd) | 1.6634(0.71sd) | 1.5245(0.64) |

(Appendix B *continued*)

**Table 4: Results of cluster analysis: Prenatal and postnatal medication received**

| Variables | Cluster 1 | Cluster 2 | Total |
| --- | --- | --- | --- |
|  | N= 1367 | N=780 | N=2147 |
|  | n (%) | n (%) | n (%) |
| Took SP/Fansider/malafan at pregnancy   - No - Yes | 250(18.29%)  1117(81.71%) | 780 (100%)  0(0.0%) | 1030(47.98%)  1117(52.03%) |
| Took chloroquine against malaria at pregnancy   - No - Yes | 1209(88.44%)  158(11.56%) | 780(39.2%)  0(0.0%) | 1989(92.64%)  158(7.36%) |
| Took proquanil against malaria at pregnancy   - No - Yes | 1365(99.85%)  2(0.15%) | 780(100%)  0(0.0%) | 2145(99.90%)  4(0.20%) |
| Took daraprim against malaria at pregnancy   - No - Yes | 1363(99.71%)  4(0.29%) | 780(100%)  0(0.0%) | 2143(99.81%)  4(0.20%) |
| Took no drug for malaria prevention at pregnancy   - No - Yes | 1367(100%)  0(0.0%) | 0(0.0%)  780(100%) | 1367(63.67%)  780(36.33%) |
| Took other drug for malaria prevention at pregnancy   - No - Yes | 1367(100%)  0(0.0%) | 256(32.82%)  524(67.18%) | 1623(75.59%)  524(24.41%) |
| Took drug for malaria prevention but don’t know name   - No - Yes | 1367(100%)  0(0.0%) | 152(19.49%)  256(32.80%) | 1519(70.75%)  256(11.92%) |
| Took drugs against intestinal parasites at pregnancy   - Don’t know - No - Yes | 57(4.12%)  722(52.82%)  588(43.01%) | 42(5.38%)  560(71.79%)  178(22.82%) | 99(4.61%)  1282(59.71%)  766(35.68%) |
| Receive Vitamin A 2 months after delivery   - No - Yes | 11(0.80%)  1356(99.19%) | 12(1.54%)  768(98.46%) | 23(1.07%)  2124(98.93%) |
| Given or bought iron tablets at pregnancy   - Don’t know - No - Yes | 12(0.88%)  119(8.71%)  1236(90.42%) | 10(1.28%)  193(24.74%)  577(73.97%) | 22(1.02%)  312(14.53%)  1813(84.44%) |

(Appendix B *continued*)

**Table 5: Results of cluster analysis: all variables in analysis**

|  | Cluster 1 | Cluster 2 | Cluster 3 | Total |
| --- | --- | --- | --- | --- |
|  | N=1168 | N=560 | N=419 | N=2147 |
|  | n (%) | n (%) | n (%) | n (%) |
| Attended to by no one during the last antenatal period   - No response - No - Yes | 0(0.0%)  1168(100%)  0(0.0%) | 0(0.0%)  560(100%)  0(0.0%) | 2(0.48%)  335(79.95%)  82(19.57%) | 2(0.09%)  2063(96.09%)  82(3.82%) |
| Attended to by physician   - No response - No - Yes | 0(0.0%)  877(75.01%)  291(24.91%) | 0(0.0%)  462(82.50%)  98(17.5%) | 2(0.48%)  340(81.15%)  77(18.38%) | 2(0.09%)  1679(78.20%)  466(21.70%) |
| Attended to by nurse/midwife   - No response - No - Yes | 0(0.0%)  220(18.84%)  948(81.16%) | 0(0.0%)  134(23.93%)  426(76.01%) | 2(0.48%)  143(34.13%)  247(58.95%) | 2(0.09%)  497(23.15%)  1621(75.50%) |
| Attended to by auxiliary staff   - No response - No - Yes | 0(0.0%)  1082(92.64%)  86(7.36%) | 0(0.0%)  523(93.39%)  37(6.61%) | 2(0.48%)  401(95.70%)  16(3.82%) | 2(0.09%)  2006(93.43%)  139(6.47%) |
| Attended to by CHO   - No response - No - Yes | 0(0.0%)  1051(89.98%)  117(10.02%) | 0(0.0%)  501(89.46%)  59(10.54%) | 2(0.48%)  385(91.89%)  32(7.64%) | 2(0.09%)  1937(90.22%)  208(9.69%) |
| Attended to by trained TBA   - No response - No - Yes | 0(0.0%)  1160(99.32%)  8(0.68%) | 0(0.0%)  558(99.64%)  2(0.36%) | 2(0.48%)  413(98.57%)  4(0.95%) | 2(0.09%)  2131(99.25%)  14(0.65%) |
| Attended to by untrained TBA   - No response - No - Yes | 0(0.0%)  1160(99.32%)  8(0.68%) | 0(0.0%)  558(99.64%)  2(0.36%) | 2(0.48%)  412(98.33%)  5(1.22%) | 2(0.09%)  2130(99.21%)  15(0.70%) |
| Attended to by village volunteer   - No response - No - Yes | 0(0.0%)  1163(99.57%)  5(0.43%) | 0(0.0%)  558(99.64%)  2(0.36%) | 2(0.48%)  417(99.52%)  0(0.0%) | 2(0.09%)  2138(99.58%)  7(0.33%) |
| Attended to by Traditional attendant   - No response - No - Yes | 0(0.0%)  1163(99.57%)  5(0.43%) | 0(0.0%)  557(99.46%)  3(0.54%) | 2(0.48%)  415(99.05%)  2(0.48%) | 2(0.09%)  2135(99.44%)  10(0.47%) |
| Attended to by other person   - No response - No - Yes | 0(0.0%)  1166(99.83%)  2(0.17%) | 0(0.0%)  558(99.64%)  2(0.36%) | 2(0.48%)  416(99.28%)  1(0.24%) | 2(0.09%)  2140(99.67%)  5(0.23%) |
| Informed where to go with any complications   - Don’t know - No - Yes | 290(24.83%)  57(4.88%)  821(70.29%) | 235(41.96%)  29(5.18%)  296(52.86%) | 212(51.84%)  17(4.06%)  190(45.35%) | 737(34.32%)  103(4.80%)  1307(60.88%) |
| Informed about signs of pregnancy complications   - Don’t know - No - Yes | 1(0.09%)  288(24.66%)  879(75.26%) | 0(0.0%)  235(41.97%)  325(58.03%) | 85(20.29%)  127(30.31%)  207(49.40%) | 86(4.01%)  650(30.27%)  1411(65.72%) |
| Offered test as part of ANC Visits   - Don’t know - No - Yes | 0(0.0%)  617(52.83%)  551(47.17%) | 0(0.0%)  389(69.46%)  171(30.53%) | 419(100%)  0(0.0%)  0(0.0%) | 419(19.52%)  1006(46.86%)  722(33.63%) |
| AIDS test done during ANC visits   - Don’t know - No - Yes | 0(0.0%)  693(59.33%)  475(40.67%) | 0(0.0%)  420(75.0%)  140(25.0%) | 415(99.05%)  4(0.95%)  0(0.0%) | 415(19.33%)  1117(52.02%)  615(28.64%) |
| Talk on MTCT –AIDS received   - Don’t know - No - Yes | 0(0.0%)  308(26.37%)  860(73.63%) | 0(0.0%)  221(39.46%)  339(60.52%) | 417(99.52%)  0(0.0%)  2(0.48%) | 417(19.42%)  529(24.64%)  1201(55.94%) |

(Appendix B *continued*)

**Table 5: Results of cluster analysis: all variables in analysis** *(continued)*

|  | Cluster 1 | Cluster 2 | Cluster 3 | Total |
| --- | --- | --- | --- | --- |
|  | N=1168 | N=560 | N=419 | N=2147 |
|  | n (%) | n (%) | n (%) | n (%) |
| Place of delivery   - Home - Public/government facility - Private facility - Other | 413(35.36%)  647(55.39%)  106(9.08%)  2(0.17%) | 286(47.86%)  224(40.0%)  45(8.03%)  5(0.89%) | 220(52.51%)  174(41.51%)  21(5.01%)  4(0.95%) | 919(42.80%)  1045(48.67%)  172(8.01%)  11(0.51%) |
| Was delivery by caesarean section   - No response - No - Yes | 1(0.09%)  1077(92.21%)  90(7.71%) | 1(0.18%)  537(95.89%)  22(3.93%) | 0(0.0%)  393(93.79%)  26(6.36%) | 2(0.09%)  2007(93.48%)  138(6.43%) |
| Timing of first check after delivery   - Immediately - Within hours - Within days - Within weeks - Don’t remember | 267(22.86%)  527(45.12%)  159(13.61%)  47(4.02%)  228(19.52%) | 71(12.68%)  195(34.82%)  86(15.36%)  27(4.82%)  181(32.32%) | 52(12.41%)  150(35.80%)  59(14.08%)  25(5.97%)  133(31.74%) | 390(18.16%)  872(40.61%)  274(12.76%)  99(4.61%)  542(25.24%) |
| Health check before discharge   - No - Yes | 465(39.81%)  703(60.19%) | 321(57.32%)  239(42.68%) | 240(57.28%)  179(42.72%) | 1026(47.79%)  1121(52.21%) |
| Took SP/Fansider/Malafan   - No - Yes | 190(16.27%)  978(83.73%) | 560(100%)  0(0.0%) | 280(66.83%)  139(33.17%) | 1030(47.97%)  1117(52.03%) |
| Took chloroquine against malaria   - No - Yes | 1046(89.55%)  122(10.45%) | 560(100%)  0(0.0%) | 383(91.41%)  36(8.80%) | 1989(92.64%)  158(7.36%) |
| Took proquanil against malaria   - No - Yes | 1166(99.83%)  2(0.17%) | 560(100%)  0(0.0%) | 419(100%)  0(0.0%) | 2145(99.90%)  2(0.09%) |
| Took daraprim against malaria   - No - Yes | 1165(99.74%)  3(0.26%) | 560(100%)  0(0.0%) | 418(99.76%)  1(0.24%) | 2143(99.81%)  4(0.19%) |
| Took no drug for malaria prevention   - No - Yes | 1168(100%)  0(0.0%) | 1(0.18%)  559(99.82%) | 198(47.26%)  221(52.74%) | 1367(63.67%)  780(36.33%) |
| Took other drug for malaria prevention   - No - Yes | 1168(100%)  0(0.0%) | 201(35.89%)  359(64.11%) | 254(60.62%)  165(39.38%) | 1623(75.59%)  524(24.41%) |
| Took drug for malaria (don’t know name)   - No - Yes | 1168(100 %)  0(0.0%) | 360(64.29%)  200(35.71%) | 363(86.63%)  56(13.37%) | 1891(88.08%)  256(11.92%) |
| Took drugs against intestinal parasites   - Don’t know - No - Yes | 45(3.85%)  616(52.74%)  507(43.41%) | 34(6.07%)  392(70.0%)  134(23.93%) | 20(4.77%)  274(65.39%)  125(29.83%) | 99(4.61%)  1282(59.71%)  766(35.68%) |
| Receive Vitamin A 2 months after delivery   - No - Yes | 8(0.68%)  1160(99.32%) | 8(1.43%)  552(98.57%) | 7(1.67%)  412(98.33%) | 23(1.07%)  2124(98.93%) |
| Given or bought iron tablets at pregnancy   - Don’t know - No - Yes | 7(0.60%)  92(7.88%)  1069(91.53%) | 9(1.61%)  106(18.93%)  445(79.46%) | 6(1.42%)  114(27.21%)  299(71.36%) | 22(1.02%)  312(14.53%)  1813(84.44%) |
|  | Mean (SD) | Mean (SD) | Mean (SD) | Mean (SD) |
| Number of ANC visits at pregnancy | 3.8014(0.54) | 3.5750(0.75) | 3.1527(1.24) | 3.6157(0.82) |
| Months before first ANC visit | 1.4118(0.54) | 1.5518(0.61) | 1.8019(0.81) | 1.5245(0.64) |

**APPENDIX C**

**Model summary and cluster quality of membership variables**


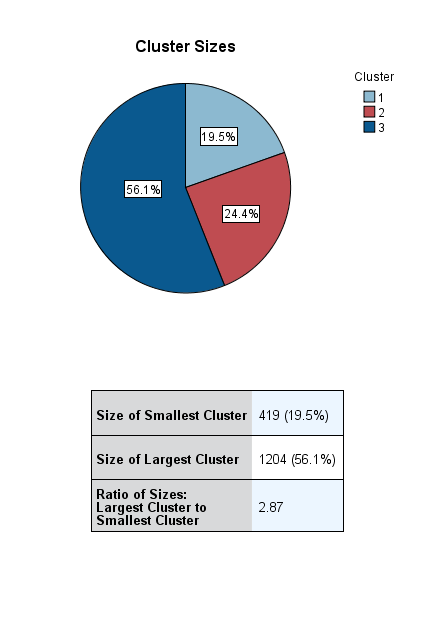
Prenatal and postnatal care


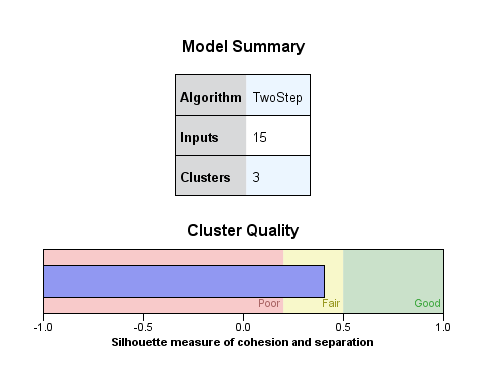


Facility Utilization and care


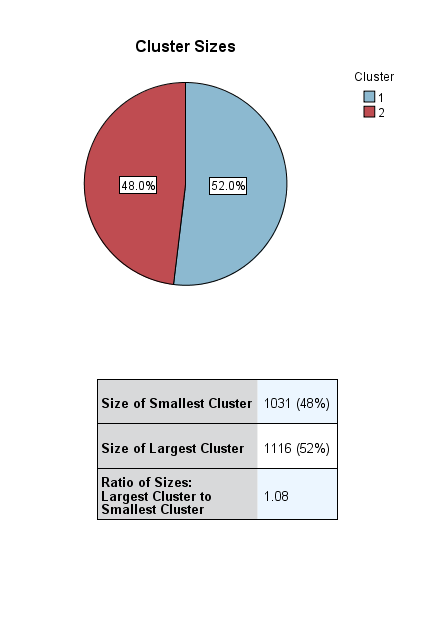

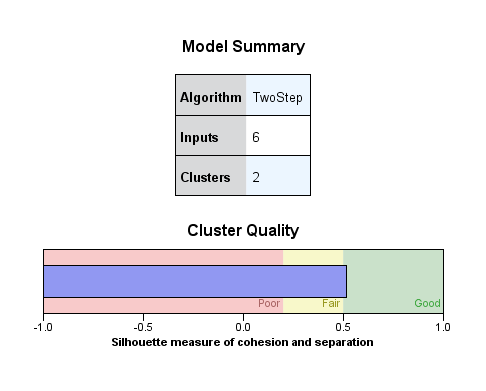


**(Appendix C *continued)***

Prenatal and postnatal care medication and care


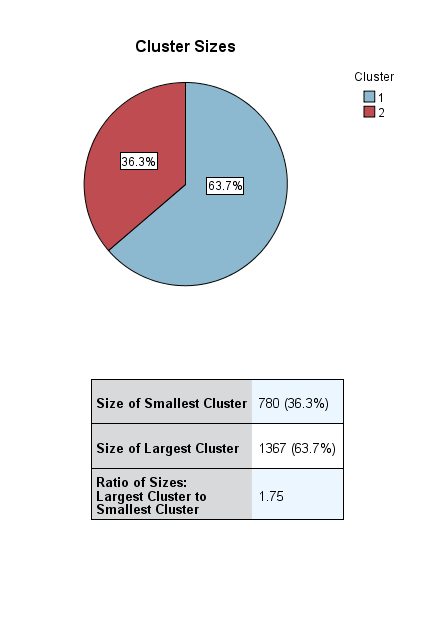


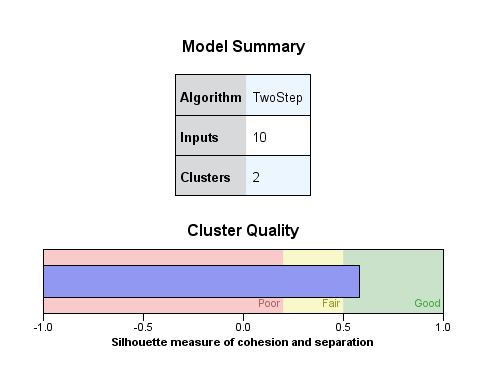


All response variables in cluster analysis


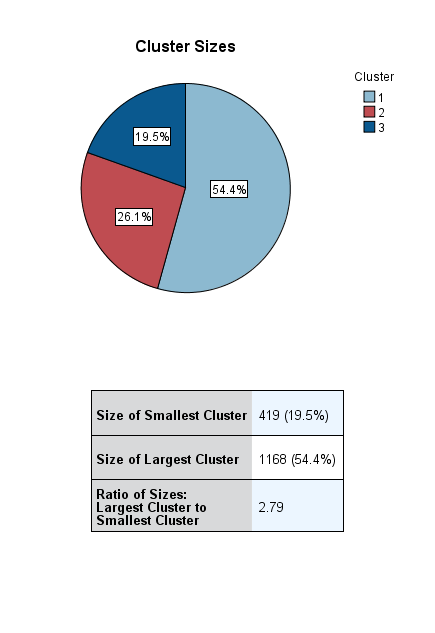

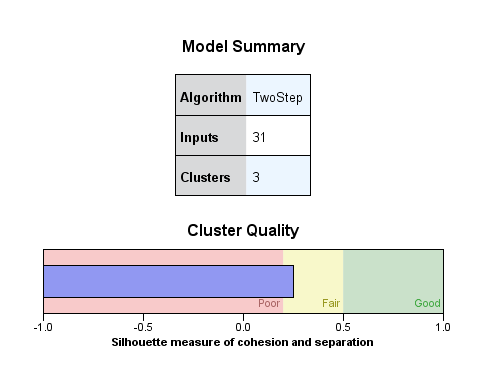

Supplement: Additional file 1: — Single and group cluster model summaries and cluster quality of membership variables. (DOCX 152 kb) [file 12913_2016_1654_MOESM1_ESM.docx]
